# BET, FTIR, and RAMAN characterizations of activated carbon from waste oil fly ash

**DOI:** 10.3906/kim-1909-20

**Published:** 2020-04-01

**Authors:** Rizwan ALI, Zaheer ASLAM, Reyad A. SHAWABKEH, Anam ASGHAR, Ibnelwaleed A. HUSSEIN

**Affiliations:** 1 Chemical Engineering Department, University of Engineering and Technology, Lahore Pakistan; 2 Department of Chemical Engineering, University of Jordon, Amman Jordon; 3 Gas Processing Center, Qatar University, Doha Qatar

**Keywords:** Activated carbon, oil fly ash, physicochemical activation, surface energy, waste utilization

## Abstract

Activated carbon (AC), a porous material with high pore volume, attracts increasing attention owing to its potential applications in several fields. The development of a porous structure in AC marginally relies on both the treatment methods and the type of precursor. Thus far, both renewable and nonrenewable precursor sources have been used to synthesize AC with high surface area and pore volume. This study presents the synthesis of AC via physicochemical treatment of waste oil fly ash (OFA), a waste material produced from power plants. The aim was to produce AC by adding surface pores and surface functional groups to the basal plane of OFA. Toward this objective, OFA was first chemically leached/activated with various combinations of H_2_SO_4_ and H_3_PO_4_, and then physically activated with CO_2_ at 900 °C. The chemical activation step, synergistically combined with CO_2_ activation, resulted in an increase of 24 times the specific surface area of the OFA. The maximum increase in surface area was obtained for the sample physicochemically treated with 100% H_2_SO_4_ . Moreover, the spectroscopic analysis confirmed the presence of acid functional groups after the chemical treatment step. To explore the surface heterogeneity, adsorptive potential distribution in terms of surface energy was also discussed as a function of the surface coverage. Following chemical activation, the OFA surface became heterogeneous. A major portion of the AC showed surface energy in the range of 40–50 erg/K, which was further increased as a result of physical activation at a higher temperature. Thus, the synergism created by physicochemical activation resulted in a material with high surface area and pore volume, and excellent adsorption characteristics. From the findings of this study, it was concluded that OFA is a cost-effective and environmentally benign precursor for the synthesis of AC.

## 1. Introduction

Activated carbon (AC), as defined by the International Union of Pure and Applied Chemistry (IUPAC), is a well-developed porous structure with several surface functionalities [1]. AC plays a contributory role in addressing the current demands of adsorbents for various applications because of its fine characteristics, like large surface area, chemical inertness, thermal stability, and mechanical strength. ACs are synthesized from various nonrenewable and renewable precursors, such as coal, petroleum pitch, lignite, lingo-cellulosic materials, or municipal wastes by physical activation or chemical treatment[2,3]. The physical activation, a 2-step procedure, involves thermochemical conversion of the precursor to char, followed by its activation using an oxidizing gas (CO_2_ , steam, or air) at higher temperatures [4–10]. On the other hand, chemical treatment involves impregnation of the precursor with a chemical agent, followed by thermal activation at an elevated temperature. Therefore, the type of chemical activating agent plays a significant role in refining the structure of carbon materials. For instance, chemical activation using different metal salts and oxidizing agents, like FeCl_3_ , zinc chloride (ZnCl_2_) , potassium hydroxide (KOH), ammonium hydroxide (NH_4_OH), phosphoric acid (H_3_PO_4_) , and HCl, leads to the pyrolysis of raw material, which allows the synthesis of AC with high carbon content, tunable porosity, and surface chemistry [11–17]. Thus far, KOH has been reported to produce high microporosity and enhance OH functional groups. The effectiveness of KOH in comparison to other chemical agents is attributed to the ability of K to intercalate the carbon easily [18,19]. A study of the literature revealed that the choice of good precursor is as important as the activating agent or treatment method in the synthesis process [20]. To date, both renewable and nonrenewable precursor sources have practiced the synthesization of AC with high surface area and pore volume. A cost-effective and environmentally friendly approach for the synthesis of AC is the use of a precursor that is not only cheap, but also environmentally benign. In this regard, palm seeds [21], oil palm shell [22], asphalt [23], oil shale [24], and many other industrial effluents, such as black liquor lignin [25], corncob [26], and fly ash [3,27,28], have been extensively explored for the synthesis of AC [29–32].

In this study, oil fly ash (OFA), which is a waste material produced from power plants, was selected as a precursor for the synthesis of AC. Several researchers have also reported the chemical activation of OFA using different chemicals, such as KOH, NH_4_OH, ZnCl_2_ , and H_3_PO_4_, followed by CO_2_ and/or steam activation at elevated temperatures [9]. OFA has high carbon contents and it can be an attractive and inexpensive precursor for the synthesis of AC. Being pozzolanic, OFA contains a high percentage of unburned carbon (~80%) along with inorganic oxides, like Fe_2_O_3_ , SiO_2_ , CaO, and Al_2_O_3_, and a trace amount of some heavy metals [33]. Common practices include the disposal of ash into ash ponds or landfills, which may cause disposal problems. In addition, leaching of trace elements from fly ash results in the contamination of aqueous streams [34,35]. This incurs economic glitches in addition to the environmental problems associated with waste disposal.

Estimates indicate that over 750 million tons of fly ash is produced globally from different sources and 50% of that is utilized in different applications, such as the synthesis of zeolite, manufacturing of ceramics and bricks, soil amendment, alumina and silica recovery, and speciation of expensive metals and as a catalyst support. At present, approximately 70–80 million tons of OFA is produced per annum from oil-based power plants in Saudi Arabia, which consume around 290–320 million barrels of crude oil as raw material [36–39]. The presence of a large amount of carbon content has attracted the attention of researchers toward the development of schemes to convert the fly ash into AC. The aim herein was to produce AC by inducing surface pores and surface functional groups to the basal plane of OFA. To achieve this objective, OFA was modified by following a 2-step treatment method: 1) chemical modification using different compositions of acid mixtures and 2) physical activation of the material synthesized in step 1 using CO_2_ under elevated temperatures. For the first step, acid mixtures comprising different combinations of H_2_SO_4_ and H_3_PO_4_were used. Activation with H_3_PO_4_alone is gaining prominence due to its ability of grafting of carbon-oxygen surface functional groups [40–43]. In contrast, other mineral acids, like HNO_3_ , H_2_SO_4_ , citric acid, and HCl, are less effective for inducing organic functional groups but more useful for producing pores on carbon substrate [44]. It has been suggested that treatment of a carbon precursor with a combination of sulfuric and phosphoric acid could produce a product with a higher proportion of micropores and cavities on the external surfaces along with different surface functional groups [45]. The change in porosity of raw OFA and modifications in the surface functional groups were analyzed by porosity and spectroscopic analyzers.

## 2. Experimental

### 2.1. Materials

OFA, acquired from the Rabigh Power plant, in Saudi Arabia, was used as the raw material. The main constituents of the raw OFA (determined by spot analysis using an energy dispersive spectrophotometer (EDX)) are presented in Table 1. The OFA has mainly carbon (~80%) in its composition, along with alkali, alkaline, and transition metals as minor elements. Analytical grade sulfuric acid (95%–98%, 1.840 g/mL) and orthophosphoric acid (assay ≥85%, 1.685 g/mL) were procured from Sigma-Aldrich (St. Louis, MO, USA). Carbon dioxide gas (purity = 99.999%) was supplied by the Saudi Industrial Gas Company.

**Table 1:** Elemental composition of the OFA before treatment.

Element	Wt%
C	77.40
O	9.32
S	7.10
Ca	0.23
Mg	1.41
Al	0.25
V	1.29
Ni	0.68
Cu	1.70
Zn	0.40
Si	0.08
Fe	0.14

### 2.2. Preparation and characterization

#### 2.2.1. Preparation of the AC

Before activation, the raw OFA was dried overnight in an oven at 110 °C. The dried sample was then cooled to room temperature and sieved through 45-μm mesh for further use. The OFA was then treated in 2 steps: 1) chemical activation using different compositions (v/v basis) of acid mixtures, i.e. H_2_SO_4_ and H_3_PO_4_, and 2) physical activation. For chemical activation, 10 g of dried OFA was mixed with 200 mL of the acid mixtures (0%–100% v/v) in a round-bottomed flask and boiled for 4 h under total reflux conditions. After completion of the reaction, the mixtures were cooled to room temperature, filtered by repeated washing with double-distilled water, and dried as before, for 5 h. In the subsequent step, 6 g of the chemically activated samples were placed in a long stainless-steel horizontal tube with a length of 20 cm and an inner diameter of 1 cm. The tube was placed in a Lindberg/Blue M TF55035A tube furnace (Waltham, MA, USA) and the temperature was ramped up to 990 °C under a continuous flow of CO_2_ (F_in_: 1 L/min; P_in_: 2 bar). The samples were held at 990 °C for 0.5 h, before being cooled down to room temperature. The physicochemical AC samples were stored in an air-tight plastic bag for analysis. The percentage of burn off, α (wt%), in each step was calculated as follows:

(1)α=Wi-WfWi

Here, Wi is the initial mass of the sample and Wf is the final mass of the sample, after CO_2_ activation. Increasing the volume percentage of sulfuric acid in the activating mixture decreased the degree of burn-off in both the chemical and physical steps (Table 2). However, keeping it at close to half minimized the loss in mass but beyond that value, the burn-off accelerated to a higher value. This was probably because of the degree of oxidation (O/H ratio in the chemical formula of acid) of the acid mixture that increased due to the involvement of sulfuric acid in the activation mixture [46].

**Table 2:** Composition of the acid mixtures (v/v basis) used in the chemical activation and corresponding loss of mass in the ash after each activation step.

Sample ID	Percentage of acids (%)		Percentage of burn-off (%)	
	H_2_SO_4_	H_3_PO_4_	Chemical activation	Physical activation with CO_2_
AC1	0	100	7.3	12.2
AC2	20	80	3.8	9.4
AC3	40	60	0.1	0.8
AC4	60	40	11.6	7.7
AC5	80	20	15.5	8.9
AC6	100	0	20.5	3.0

#### 2.2.2. Material characterization

The surface functional groups of the raw and activated samples were analyzed using a FPC FTIR (PerkinElmer, Waltham, MA, USA). The procedure involved mixing 1.0 g of the dried sample with an equal amount of KBr powder and subjecting the resultant mixture to 10 ton/m^2^ pressure to obtain a thin transparent disk. The disk was oven-dried at 110 °C to minimize the interference with moisture. The spectra were recorded in the region of 0–4000 cm^-1^ and believed to be accurate within ±1 cm^-1^ . Similarly, the Raman spectra (RENISHAW inVia RA 802) were recorded in the same range using a single monochromatic source for spectral light. The Brunauer-Emmett-Teller (BET) surface area and pore size distribution of the raw and activated samples were determined using a Micrometrics ASAP 2020 surface and pore size analyzer (Atlanta, GA, USA). Prior to each experiment, approximately 0.4 g of the sample was taken and subjected to a degassing process at 300 °C for 3 h. Subsequently, the pore size distribution and BET surface area were measured using a nitrogen adsorption-desorption isotherm at 77 K (interpreted using the BET and Barrett, Joyner, and Halenda (BJH) methods). The surface energy distributions were extracted using the source file obtained from the Micrometrics ASAP 2020 analyzer. The energies were obtained by integrating the integral equation of adsorption, where the quantity adsorbed per unit area at some pressure on a surface had the same energy.

## 3. Results and discussion

### 3.1. Spectroscopic analysis

**Figure 1:**
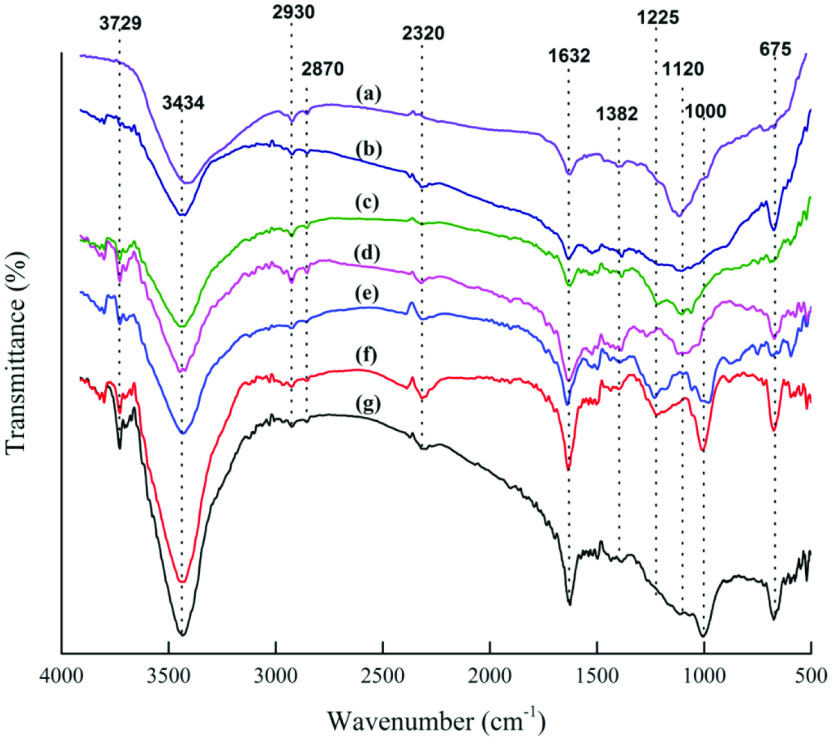
FTIR spectra of the untreated and chemically activated OFA: (a) raw, OFA, (b) AC6, (c) AC5, (d) AC4, (e) AC3, (f) AC2, and (g) AC1.

Figure 1 compares the FTIR spectra of the raw OFA and physicochemical AC samples. The infrared transmission peak recorded at a wavelength range of 3200–3650 cm^-1^ exhibited a wide and broad transmission band with its maxima at 3440 cm^-1^ . Here, the presence of a broad transmission band was attributed to the O-H stretching mode of hydrogen-bonded hydroxyl groups attached to the structure or surface-adsorbed moisture [45,47]. The peak intensity decreased with an increase in the H_2_SO_4_ concentration, and thus indicated the dehydrating nature of H_2_SO_4_ . However, the appearance of wider peaks at lower wavenumbers endorsed the presence of strong hydrogen bonding [48]. For the physicochemically activated samples, a transmission band with a low signal appeared at around 3729 cm^-1^ . Ascribed to free O-H functional groups, the peak intensity decreased with the H_2_SO_4_ concentration, as observed in Figure 1. In addition, 2 consecutive small peaks at around 2900 and 2810 cm^-1^ , which appeared for all of the samples, were ascribed to the presence of asymmetric and symmetric C-H stretching vibrations of the hydrocarbon structure, respectively [47–49].

In Figure 1, the infrared transmission peak that appeared at 1632 cm^-1^ was attributed to C=O stretching vibrations of the conjugated hydrocarbon. This signal was weak for the raw OFA sample. Following acid treatment, the signal gained strength and centered around 1625–1639 cm^-1^ due to the combined effect of the carbonyl functional group C=O and C=C stretching vibrations of the aromatic rings [50–52]. Implying the oxidative nature of orthophosphoric acid, the peak intensity increased with an increase in the H_3_PO_4_concentration due to the formation of oxygen- and/or phosphorus-containing functional groups [45,53]. In addition, a weak signal associated with the asymmetric stretching of S=O appeared at around 1382 cm^-1^ [54]. This peak was stronger in the AC6 sample and decreased with a decrease in the H_2_SO_4_ concentration in the acid mixture. A broad transmission band at around 1000–1300 cm^-1^ commonly existed in the spectra of the oxidized carbons and was attributed to stretching of the C-O group from alcohol, acids, phenols, ethers, and/or ester functional groups. It was also a characteristic peak for the phosphorous and phosphocarbonaceous compounds present in the phosphoric acid-activated surfaces. Nevertheless, the transmission bands in this range may have overlapped due to the presence of both oxygen- and phosphorous-containing functional groups, and thus made it difficult to confirm the presence of a particular functional group.

The peak that appeared at around 1180–1280 cm^-1^ was ascribed to the stretching mode of the hydrogenbonded P=O. As per the literature, it may have also represented the O-C stretching mode of the P-O-C (aromatic) or P=OOH groups [49]. This peak was present in all of the activated samples, but decreased with an increase in the H_2_SO_4_ concentration. A weak signal against conjugated C-O stretching appeared at 1118 cm^-1^ . The signal was strong in the raw OFA and varied within the transmission wavelength range of 1100–1000 cm^-1^ with a change in the acid composition [45]. In samples AC1, AC2, and AC3, the peak at 1000 cm^-1^ was associated with ionized linkage P+–O¯in the acid phosphate esters or symmetric vibrations in the chain of P-OP (polyphosphate). It may also have been linked with P-O-C (aliphatic and aromatic) asymmetric stretching, P–O stretching in ?P=OOH, P–OH bending, P–O–P asymmetric stretching in polyphosphates or symmetric stretching of PO2 and PO3 in phosphate-carbon complexes [49]. However, the peak intensity was observed to show a decreasing trend with an increase in the H_2_SO_4_ concentration. In addition, a shift from 1000 cm^-1^ to 1120 cm^-1^ was observed in samples AC4, AC5, and AC6 due to S=O stretching vibrations of the sulfates and sulfoxides. For the H_2_SO_4_ -treated sample (AC6), a peak indicating S-O stretching vibration appeared in the lower regions, 673 cm^-1^ , due to the presence of sulfuric acid surface functional groups. The peak position was further shifted to lower regions when the raw OFA was treated with an acid mixture containing a higher concentration of H_3_PO_4_, as observed in the case of samples AC2, AC3, AC4, and AC5. It was probably due to the presence of P=S and P-C stretching of organophosphorus compounds, which may superimpose the outof- plane deformation vibrations of C-H in different aromatic structures [54–56]. However, the appearance of a small peak below 600 cm^-1^ confirmed the presence of orthophosphate salt, sulfanylidene phosphane ion, and P+-O¯ ionized linkage (deformation mode), due to the rich content of orthophosphoric acid in the activated samples [55].

**Figure 2:**
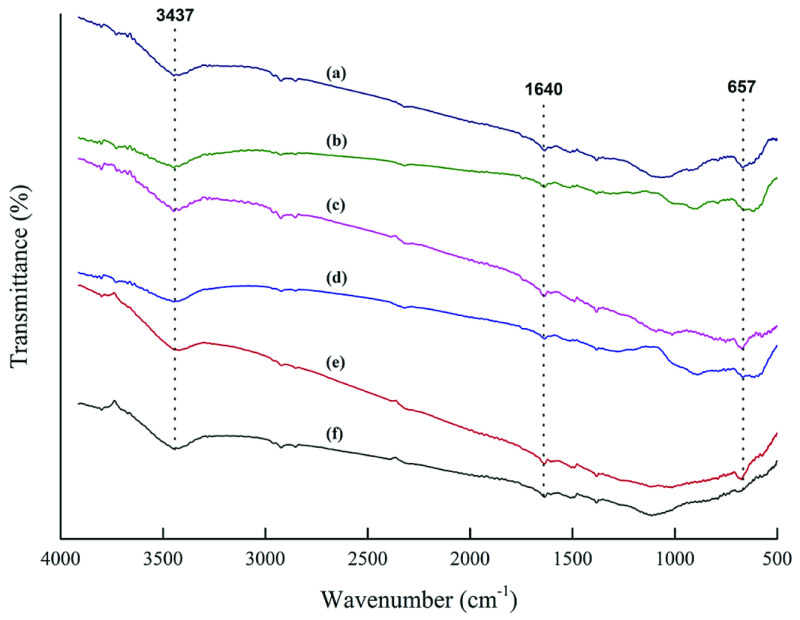
FTIR spectra of the physicochemically activated OFA: (a) AC6, (b) AC5, (c) AC4, (d) AC3, (e) AC2, and (f) AC1.

The physicochemical activation of the raw OFA resulted in the removal of most of the carbonyl functional groups. As observed in Figure 2, two small peaks, indicating the stretching vibration mode of hydrogen-bonded O-H functional groups and C=C stretching, appeared at around 3400 cm^-1^ and 1600 cm^-1^ , respectively [45,47,50,51]. In addition, a broad band associated with the coupled vibrations of C-H bending in the carbon structure and physiosorbed CO_2_ appeared at around 618–676 cm^-1^ . This implied that physical activation caused some surface changes and resulted in the removal of oxygen-containing functional groups, as observed in Figure 2 [54].

**Figure 3:**
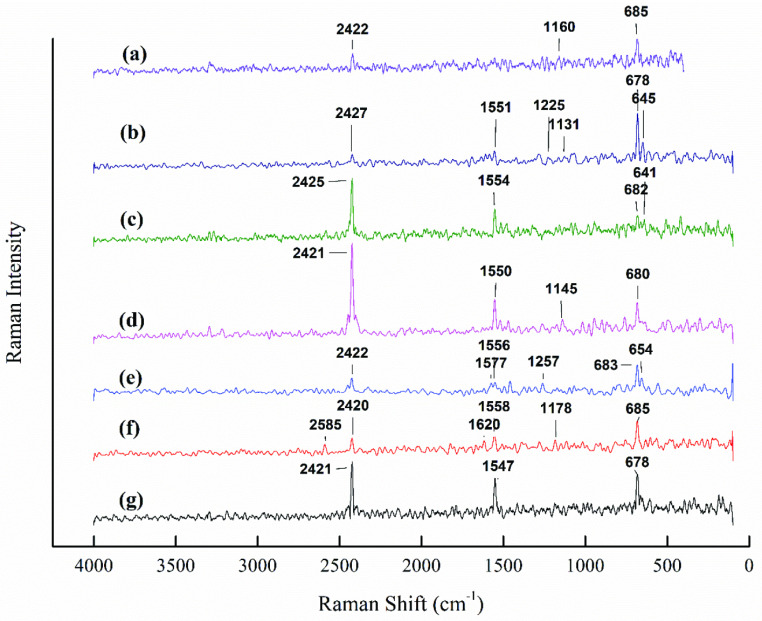
Raman spectra of the untreated and acid-treated OFA without CO_2_ activation: (a) raw OFA, (b) AC6, (c) AC5, (d) AC4, (e) AC3, (f) AC2, and (g) AC1.

In order to support the infrared spectroscopic analysis, chemically activated samples were examined through Raman spectroscopy and the results are presented in Figure 3. Consistent with the FTIR results, the Raman spectra were observed to show a shift within the band range of 600–700 cm^-1^ . Like the FTIR results, the shift in the Raman spectra was attributed to out-of-plane vibrations of the C-H bond in different aromatic structures. It may also have correspond to strong stretching of S-O, P=S, and P-C, due to the formation of sulfuric surface groups, phosphorous pentasulfide, and organophosphorus compounds, respectively [54–56]. In addition, small peaks appearing at around 1100–1300 cm^-1^ corresponded to S=O, P=O, and P–O vibrations due to the presence of organic sulphates and phosphorous compounds in the chemically activated OFA samples. A peak at around 1160 cm^-1^ was ascribed to C-O-C stretching vibrations in the acid-treated samples. Inconsistent with the FTIR, a new Raman peak appeared at around 1500 cm^-1^ in all of the acid-treated OFA samples. Ascribed to C-C and C=C stretching vibrations of the aromatic rings, the peak intensity increased with an increase in the H_3_PO_4_concentration in the acid mixture. In addition, a sharp peak at around 2420–2430 cm^-1^ was assigned to C=O stretching vibrations of the conjugated systems, such as ketoester, keto-enol, and diketone structures. The spectroscopic results showed the removal of most of the functional groups in the OFA due to the combined effect of heat and CO_2_ . From the aforementioned discussion, it was concluded that chemical activation with the acid mixtures anchored various surface functional groups on the surface of the OFA. These functional groups may have provided potential active sites for the adsorption of pollutants from aqueous/gaseous phases or the initiation of surface reactions during the thermophysical treatment. As a result, most of the surface functional groups were removed from the surface of the physicochemically treated OFA, as also confirmed from the FTIR spectra presented in Figure 2.

### 3.2. Surface area and porosity analysis

#### 3.2.1. Nitrogen adsorption/desorption isotherms

The adsorption/desorption isotherms for the raw and ACs are presented in Figures 4a–4c, where it can be observed that the raw OFA and AC samples exhibited a typical type-III-like isotherm that had a different width of the hysteresis loop. The hysteresis in the N2 adsorption/desorption isotherm was usually associated with capillary condensation in the mesoporous structure. A weak hysteresis effect can be observed in the raw OFA, as shown in Figure 4a. The AC1, AC2, AC3, and AC4 activated samples exhibited negligible hysteresis loop effects, while samples AC5 and AC6 exhibited capillary condensation accompanied by a wide hysteresis loop (Figure 4b). The curves for samples AC5 and AC6 exhibited type-H4- and Type-H3-like isotherms (associated with the tensile strength effect (TSE)), respectively. The shape of these curves indicated the presence of slittype narrow micro- and mesopores in the samples [57]. In comparison, sample AC6 was observed to show high porosity and surface area (Figure 4c), which was due to the higher oxidative affinity of sulfuric acid for the OFA surface during the chemical activation process.

**Figure 4a:**
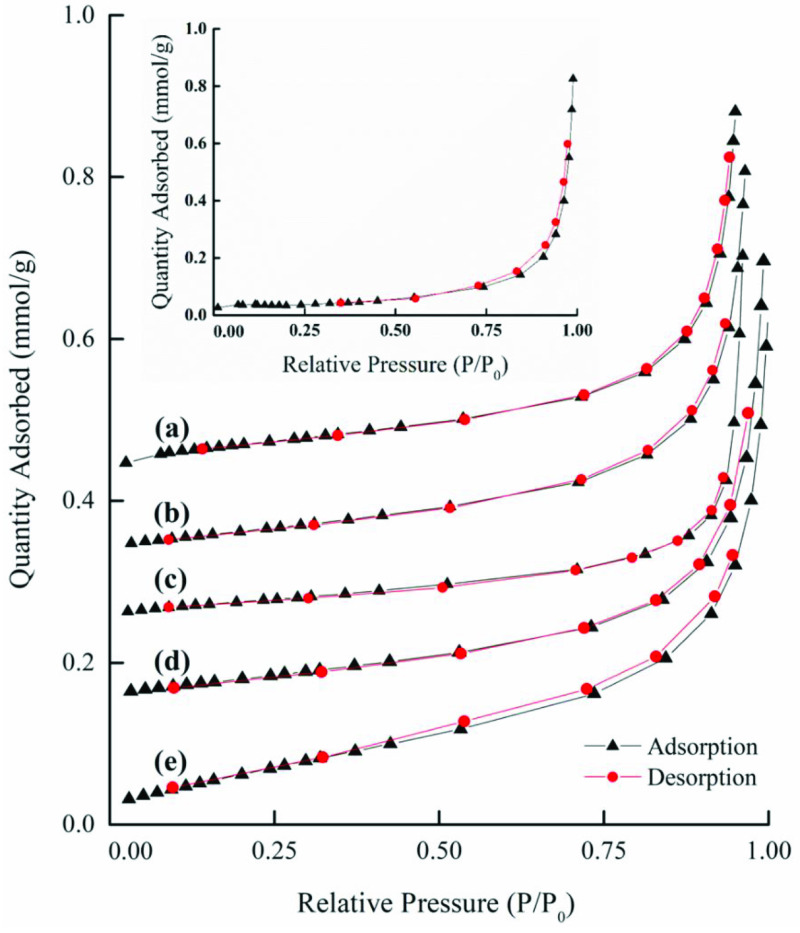
Nitrogen adsorption-desorption isotherm of the raw (inset) and chemically activated OFA: (a) AC1, (b) AC2, (c) AC3, (f) AC4, and (e) AC5.

**Figure 4b:**
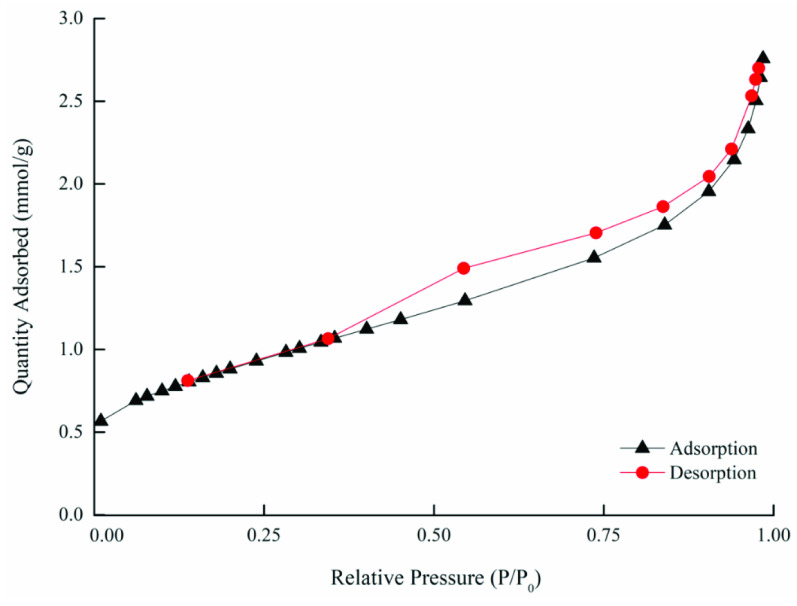
Nitrogen adsorption-desorption isotherm of AC6.

**Figure 4c:**
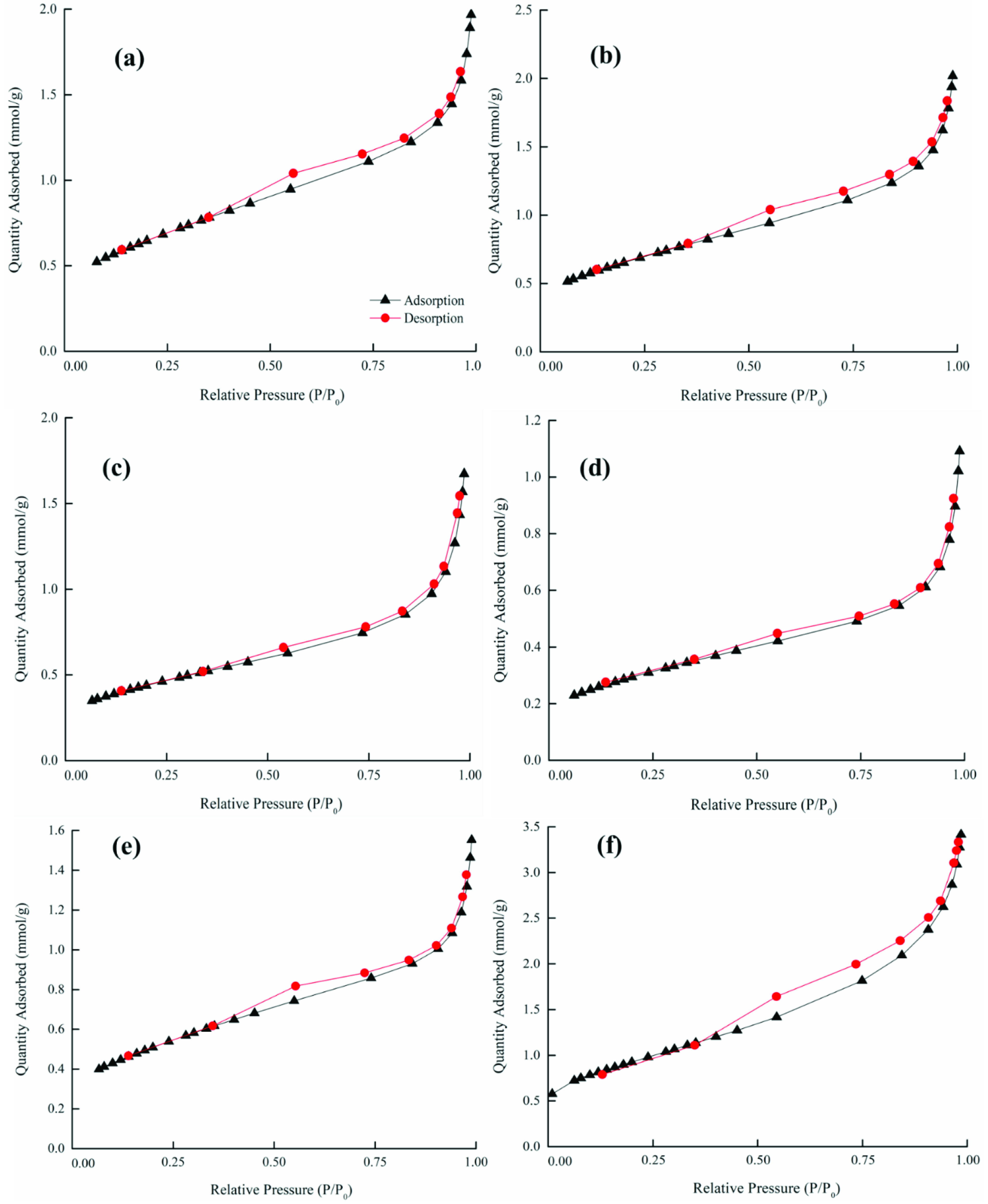
Nitrogen adsorption-desorption isotherm of physicochemically activated OFA: (a) AC1, (b) AC2, (c) AC3, (f) AC4, (e) AC5, and (f) AC6.

**Figure 5:**
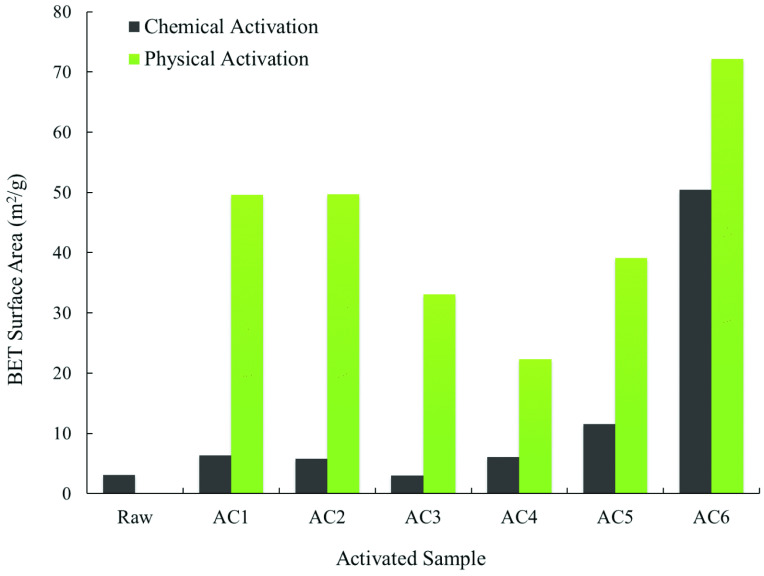
Comparison of the BET surface area of all of the ACs.

Following physical treatment, the adsorption isotherms for the chemically activated OFA exhibited pronounced hysteresis at P/Po >0.3 due to N2 capillary condensation in the mesopores (Figure 5). According to the IUPAC classification, the hysteresis of such a type conforms to a type-III isotherm (with no limiting adsorption at a high P/Po) . Thus, the hysteresis loop effects became effective in the physicochemically activated samples, indicating the presence of pores with a wider length and narrow slits. However, the desorption curve showed a strong drop in N2 adsorbed volume near P/Po = 0.37. This was probably due to the sudden transformation of liquid nitrogen into gaseous nitrogen, which is often referred to as the TSE. Here, it is pertinent to highlight two important features of the hysteresis loop. First, the steep region in the desorption curve leading to the lower closure point around a relative pressure from 0.4 to 0.3, which is termed as the force closed phenomenon. This phenomenon is typically referred to the TSE, which is attributed to instability of the meniscus condensation in the pores. Second, the unclosed hysteresis portion reflects the potential existence of slit-type narrow pores [58].

#### 3.2.2. Surface area and pore size distribution

Figure 5 compares the BET surface area of the samples obtained from the N2 adsorption/desorption isotherms. As expected, the BET surface area of the treated samples was significantly improved in comparison with 3 m^2^ /g of the raw OFA. For the physicochemically activated samples, the BET surface area increased by 2 to 8 times due to the coupled effect of chemical oxidation and high temperature treatment. Furthermore, the BET surface area revealed an increasing trend with a rise in the H_2_SO_4_ concentration in the acid mixture. As a result, sample AC6 exhibited a higher BET surface area of 50 m^2^ /g, which further increased to 70.2 m^2^ /g after physical activation. This implied that H_2_SO_4_ activation not only affected the surface area of the raw OFA, but also induced more pores during physical activation with CO_2_ .

**Figure 6:**
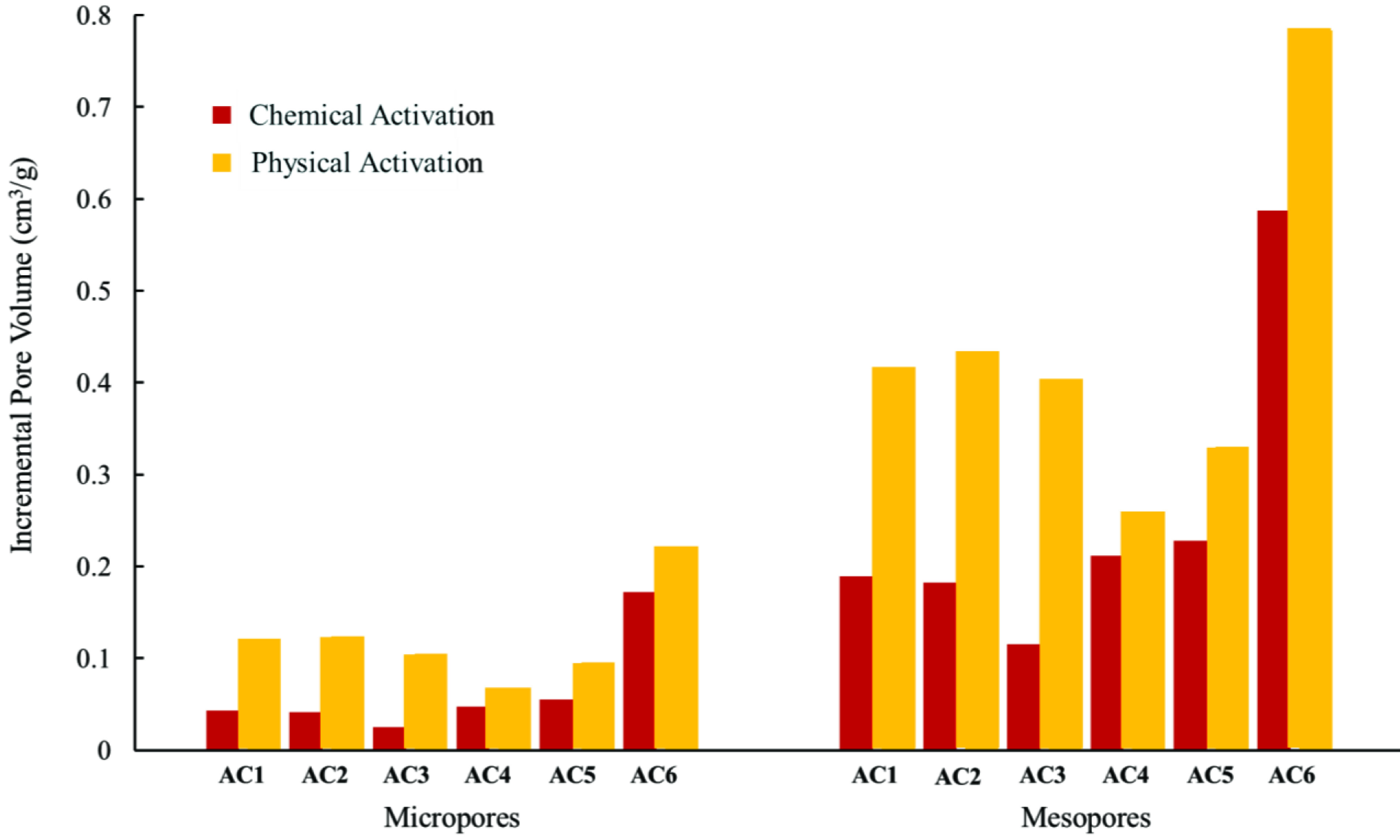
Incremental pore volume of the micropores and mesopores in all of the activated samples during physicochemical activation.

The contribution of micro- and mesopores to the total pore volume is presented in Figure 6. As observed, the mesopores constituted 60%–80% of the total pore volume, which was the highest contribution towards the pore volume. In addition, an increase in the H_2_SO_4_ :H_3_PO_4_ratio resulted in an increase of both the micro- and mesopores. Therefore, sample AC6 exhibited the highest contribution of both micro- and mesopores to the total pore volume. Furthermore, physical treatment of the chemically activated samples resulted in a further increase in pore volume due to the release of volatiles from the interior of the pores. An increase in temperature also led to a higher degree of microporosity due to the burning of carbon in the ash structure. The high surface porosity enhanced the sorption characteristics of the activated OFA samples. Subsequently, sample AC6 exhibited high adsorption capacity due to a large number of mesopores and higher surface area. The adsorption potential energy was higher in the mesopores due to the heterogeneous nature of the AC surface, interconnected pores, and the pore geometry effect of the activated ash [59].

#### 3.2.3. Surface heterogeneity of the synthesized AC

Surface energy is energy associated with the adsorption of a molecule onto a specific site on the solid surface within the pore volume [60]. Surface energy is directly linked to surface heterogeneity, which is primarily because of the presence of heteroatoms and surface functional groups. Other reasons may include structural or surface imperfections due to the presence of dislocations, holes, pits, cracks, blocked pores, or chemically bonded contaminants. In porous solids, the main source of surface heterogeneity is the complex porous structure containing micro- and mesopores [61]. Any of these factors may introduce a certain level of binding site heterogeneity. Hence, a site with high surface energy can adsorb a molecule at low pressure. However, high pressure is required to get the molecule adsorbed on a site with low surface energy.

**Figure 7:**
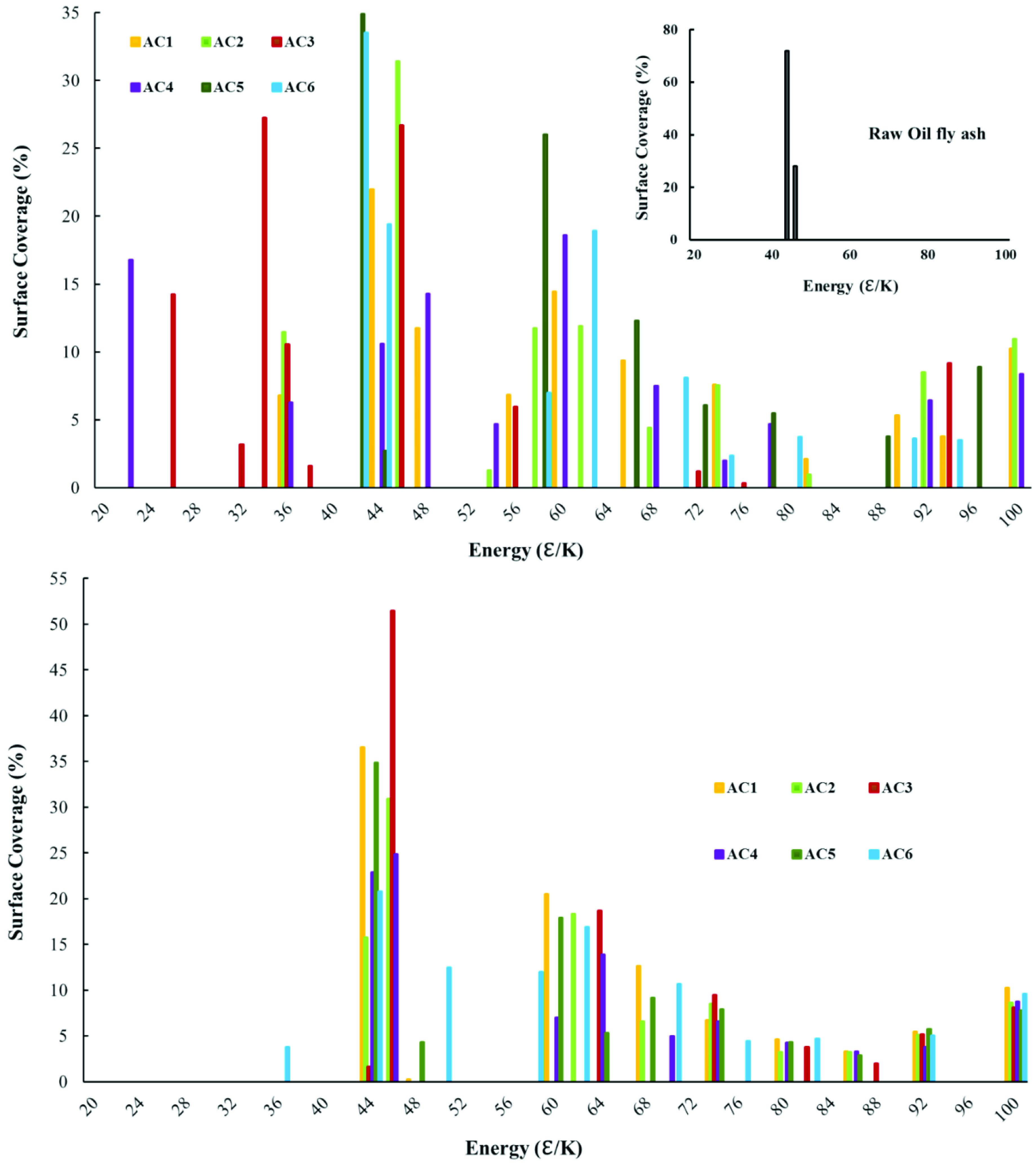
Surface energy distribution of the OFA: (a) after chemical activation and (b) after physicochemical activation.

In this study, the synthesized AC samples were characterized to determine their surface heterogeneity. The surface coverage, as a function of site energy, is presented in Figure 7a. Two prominent peaks appeared in the range of 42–48 erg/K (insert graph in Figure 7a), indicating that the raw OFA had less surface heterogeneity and a nonporous structure. However, chemical activation of the raw OFA produced a surface with high surface heterogeneity and sites with different adsorption potential. After chemical activation, samples AC3 and AC4 produced patches of low surface energy in the range of 20–50 K. However, the surface energy of all of the ACs spread over the whole range with maxima was in the range of 42–48 K, as shown in Figure 7a. Acid mixtures with a moderate degree of oxidation (samples AC3 and AC4) produced a more heterogeneous surface when compared to mixtures with a higher degree of oxidation (i.e. samples AC5 and AC6), as shown in Figure 7a. The higher degree of oxidation was responsible for the formation of high surface energy sites, as confirmed by the surface coverage distribution value, which was higher than 40 K.

Physical treatment of the ACs at elevated temperatures resulted in the removal of low-energy surface sites and a subsequent reduction in the surface heterogeneity (Figure 7b). The surface coverage of samples AC3 and AC4 shifted to higher surface energy and approximately 50% of the surface coverage was observed in the range of 42–46 K. One can easily observe the transfer of the distribution model from multimodel surface coverage distribution (Figure 7a) to bimodel distribution (Figure 7b). Multiple peaks in the chemically activated ACs could be associated with surface functional groups on the carbon surface, and the functional groups were vulnerable to heat and probably left the surface during physical activation at higher temperatures. Physical activation generated more porosity on the carbon surface, which led to less surface heterogeneity and higher adsorption energy sites for the probe molecule. The only reason for surface heterogeneity after physicochemical activation (Figure 7b) of the OFA was probably due to the different dimensions of the micro- and mesopores that offered a different surface potential to the adsorbate molecule.

## 4. Conclusion

Waste OFA is an environmentally benign and cost-effective precursor for the synthesis of AC. In this study, AC was synthesized by adding surface pores and surface functional groups to the basal plane of OFA. The OFA was then treated in two steps: 1) chemical activation using different compositions of acid mixtures, i.e. H_2_SO_4_ and H_3_PO_4_, and 2) physical activation. Acid leaching in the chemical activation step anchored sulfonic and phosphoric functional groups on the surface of the AC. The porosity analysis also showed a substantial increase in the surface area of the AC, at 24 times higher than the raw OFA (3 m^2^ g−1) . The pore size distribution also revealed that the physicochemically activated OFA was rich in mesopores, which actually contributed to ~90% of the total pore volume. The adsorption potential distribution confirmed that the surface of the AC was heterogeneous. The AC obtained after the chemical activation step had a wider distribution of site energies. A considerable percentage of the pores in samples AC3 and AC4 had site energies of less than 44 (?/K), representing the difficult adsorption of adsorbate. However, physical activation with CO_2_ at higher temperatures shifted the surface energy to higher values. The maximum of distribution function of all of the synthesized AC was above 42 (?/K), which corresponded to the easy adsorption of nitrogen on the carbon surface after physicochemical activation of the raw OFA.
